# Positive association between sodium-to-chloride ratio and in-hospital mortality of acute heart failure

**DOI:** 10.1038/s41598-024-58632-4

**Published:** 2024-04-03

**Authors:** Dongmei Wei, Shaojun Chen, Di Xiao, Rongtao Chen, Yuanting Meng

**Affiliations:** 1 Department of Cardiovascular, Liuzhou Traditional Chinese Medical Hospital, Liuzhou, 545001 China; 2 Guangxi University of Chinese Medicine, Nanning, 530000 China

**Keywords:** Sodium-to-chloride ratio, Acute heart failure, Mortality, MIMIC-IV, Heart failure, Metabolic disorders

## Abstract

Previous studies have suggested that levels of sodium and chloride in the blood may be indicative of the prognosis of different medical conditions. Nevertheless, the assessment of the prognostic significance of the sodium-to-chloride (Na/Cl) ratio in relation to in-hospital mortality among individuals suffering from acute heart failure (AHF) remains unexplored. In this study, the participants were selected from the Medical Information Mart for Intensive Care IV database and divided into three groups based on the Na/Cl ratio level upon admission. The primary results were the mortality rate within the hospital. Cox regression, Kaplan–Meier curves, receiver operator characteristic (ROC) curve analysis and subgroup analyses were utilized to investigate the correlation between the admission Na/Cl ratio and outcomes in critically ill patients with AHF. A total of 7844 patients who met the selection criteria were included in this study. After adjusting for confounders, the multivariable Cox regression analysis revealed that the baseline Na/Cl ratio significantly elevated the risk of in-hospital mortality among critically ill patients with AHF (HR = 1.34, 95% CI 1.21–1.49). Furthermore, when the Na/Cl ratio was converted into a categorical factor and the initial tertile was taken as a point of comparison, the hazard ratios (HRs) and 95% confidence intervals (CIs) for the second and third tertiles were 1.27 (1.05–1.54) and 1.53 (1.27–1.84), respectively. Additionally, a *P* value indicating a significant trend of < 0.001 was observed. ROC curve analysis showed that Na/Cl ratio had a more sensitive prognostic value in predicting in-hospital mortality of AHF than the sodium or chloride level alone (0.564 vs. 0.505, 0.544). Subgroup examinations indicated that the association between the Na/Cl ratio upon admission and the mortality rate of critically ill patients with AHF remained consistent in the subgroups of hyponatremia and hypochlorhydria (*P* for interaction > 0.05). The linear relationship between the Na/Cl ratio and in-hospital mortality in AHF patients indicates a positive association.

## Introduction

Acute heart failure (AHF) is a clinical syndrome marked by a sudden decline in cardiac output and tissue perfusion, predominantly manifesting as blood circulation congestion, that occurs rapidly and poses a serious threat to the patient’s health and life^1,2^. Despite significant advancements in pharmacological and device-based treatments for AHF, the incidence and mortality rate remain high^3^. Therefore, accurately assessing the condition of patients with AHF and correctly evaluating their prognosis, as well as promptly implementing effective treatment measures and controlling factors that can influence prognosis, hold great significance.

Due to disruptions in the renin-angiotensin system, increased levels of antidiuretic hormones, and the use of diuretics in patients with AHF, electrolyte imbalances, particularly involving sodium and chloride, are commonly observed^4^. Electrolytes play a crucial role in maintaining cardiac function and hemodynamic stability. Disruptions in serum levels of sodium and chloride can interfere with the normal electrical activity of the heart, leading to arrhythmias and impaired contractile function. These changes in electrolytes may impact fluid balance, worsen congestion, and exacerbate symptoms of heart failure (HF)^5^. Furthermore, electrolyte imbalances are associated with systemic inflammation, oxidative stress, and endothelial dysfunction, all of which are key drivers of HF progression and adverse outcomes^6,7^. Therefore, the intricate interplay between electrolyte disturbances and these pathological processes has a significant impact on the mortality of patients with AHF.

It seems to be widely acknowledged that serum sodium levels are associated with the prognosis of critically ill patients, including those with AHF^8–10^. However, there is some controversy regarding the impact of serum chloride on the prognosis of patients with HF. Some studies have indicated an independent inverse correlation between admission serum chloride levels and short-term mortality rates during hospitalization for AHF^11–13^. Other research has demonstrated that both hypochloremia and hyperchloremia are associated with adverse outcomes in patients with HF^14–16^. Sodium ions and chloride ions, the most important cations and anions in the human body, are closely interconnected and interact with each other. The results of the Tolvaptan Trial also suggest that solely focusing on serum sodium may not improve short-term and long-term prognosis in patients; thus, the impact of serum chloride needs to be considered as well^17^. Therefore, this study aims to thoroughly consider the influence of both serum sodium and chloride levels on the prognosis of patients with AHF. By analyzing the ratio of serum sodium to serum chloride (Na/Cl) in AHF patients, relevant risk factors for prognosis will be identified and a reference for individualized treatment will be provided by clinical physicians, as well as high-risk individuals with adverse prognoses being screened.

## Materials and methods

The study followed the Strengthening the Reporting of Observational Studies in Epidemiology (STROBE) reporting guidelines^18^.

### Database

The information utilized in this research was obtained from the Medical Information Mart for Intensive Care (MIMIC)-IV (Version 2.0), a well-known and easily accessible database containing clinical data from intensive care units. MIMIC-IV, an enhanced edition of MIMIC-III, includes admission data for more than 50,000 patients who were in the ICU at the Beth Israel Deaconess Medical Center in Boston, Massachusetts, between 2008 and 2019. To obtain access, author Dongmei Wei successfully completed an online training course and exam (Certification Number: 48693003). To protect the privacy of every patient, MIMIC-IV utilizes unidentified personal identifiers.

### Study population

**Figure 1:**
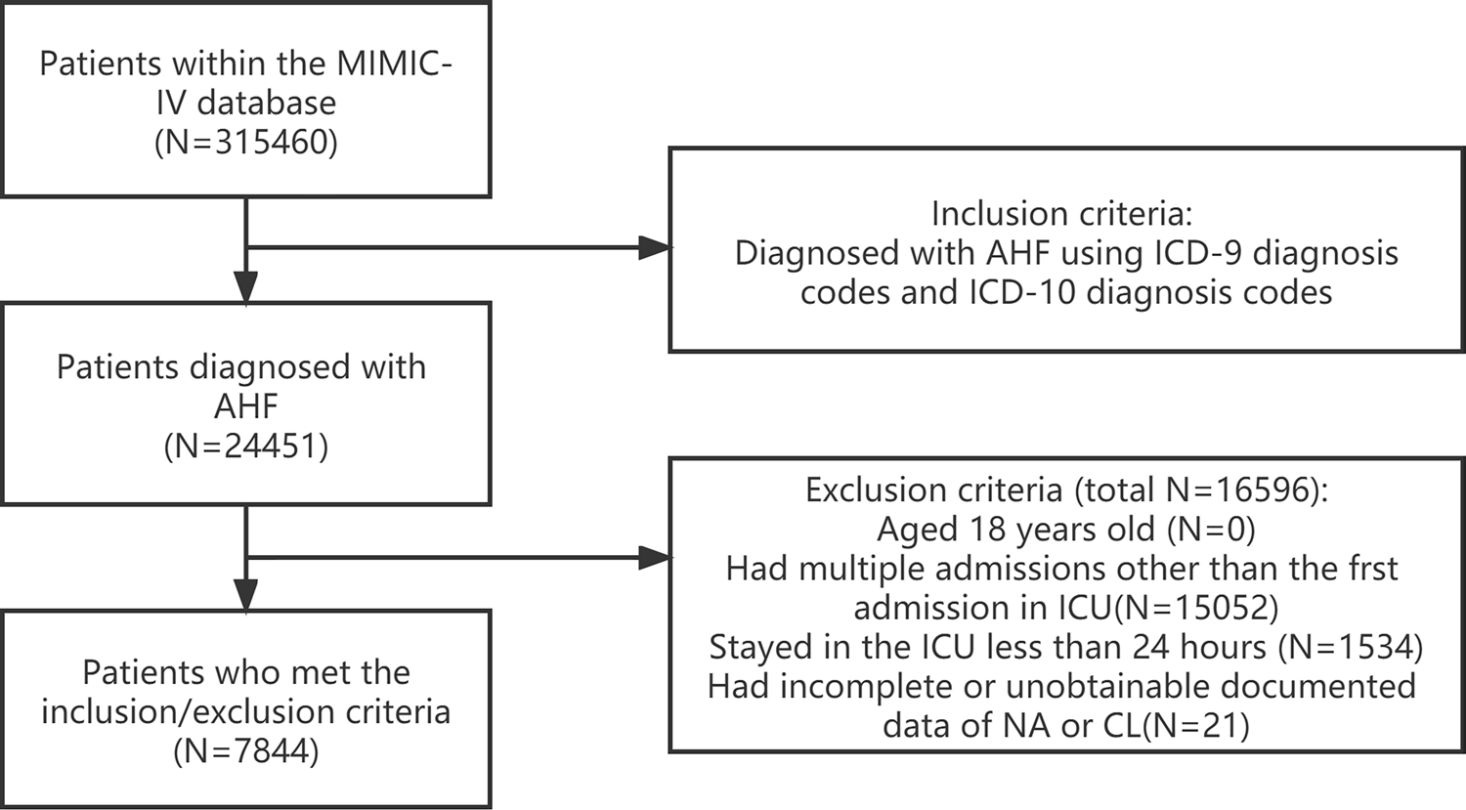
Flowchart of patient selection.

This study enrolled patients with AHF who were admitted to the intensive care unit (ICU) in the MIMIC-IV database between 2008 and 2019. The clinical diagnosis of AHF was confirmed using data from the hospital records and was based on the primary diagnosis code according to the International Classification of Diseases (ICD). Patients with AHF as the primary diagnosis, regardless of whether it was systolic HF, diastolic HF, or a combination of both, with congestive or non-congestive features, left ventricular or right ventricular failure, or acute exacerbation of chronic HF, were included in this study. The inclusion criteria for patients was the presence of a diagnosis “acute heart failure” in the long title of the diagnosis dictionary. The specific ICD codes and diagnosis names are shown in Supplementary Table 1. The exclusion criteria included (1) individuals below 18 years old, (2) individuals not admitted to the ICU, (3) individuals with an ICU stay duration of less than 24 h, (4) individuals without serum sodium or serum chloride data, and (5) individuals with multiple ICU admissions for whom only the initial ICU record was taken into account. Figure 1 depicts the process of selecting patients.

### Exposure variable

Within 24 h of patient admission, the levels of sodium and chloride in the serum were measured. The MIMIC-IV database provides a range of 135–145 mmol/L for serum sodium and 96–106 mmol/L for serum chloride. Hyponatremia (< 135 mmol/L) and hypernatremia (> 145 mmol/L) are defined by serum sodium levels, while hypochloremia (< 96 mmol/L) and hyperchloremia (> 106 mmol/L) are defined by serum chloride levels. To prevent pseudohyponatremia, we corrected serum sodium levels using the following formula: Corrected Sodium (Hillier) = Measured sodium + 0.024 × (Serum glucose − 100)^19^. The Na/Cl ratio, calculated by dividing serum sodium by serum chloride, was used to categorize patients into three tertiles based on admission levels. This categorization aimed to explore the relationship between Na/Cl ratio and in-hospital mortality in critically ill patients with AHF.

### Covariates

The initial measurements taken within the first 24 h of ICU admission were used as the measurement parameters for this study. To extract patient information, Structured Query Language (SQL) and Navicat software (version 15) were utilized to retrieve the necessary data. The variables that were extracted consisted of basic characteristics such as age and sex. Additionally, classification of AHF, type of imbalance of sodium or chloride and other health conditions, such as myocardial infarction, cerebrovascular disease, chronic pulmonary disease (COPD), liver disease, chronic kidney disease, hypertension, and diabetes, were documented. The vital signs, which included the heart rate, mean blood pressure, respiratory rate, temperature, and oxygen saturation of blood (SaO_2),_ were recorded. Additionally, data regarding the administration of diuretics, along with details about the utilization of mechanical ventilation (MV) and the current state of percutaneous coronary intervention (PCI), were gathered. The laboratory values included measurements of white blood cell count (WBC), hemoglobin, platelets, glucose, sodium, chloride, potassium, calcium, serum creatinine, blood urea nitrogen, bicarbonate, urinary sodium, urinary chloride, albumin, and NT-proBNP. The estimated glomerular filtration rate (eGFR) was determined using the Chronic Kidney Disease Epidemiology Collaboration equation (CKD-EPI)^20^. Furthermore, data on hospitalization and admission to the ICU, which encompassed the duration of hospital stays, ICU stays, mortality rates within the hospital, and acute kidney injury (AKI) within 2 days or 7 days, were documented. Scoring systems such as the Charlson comorbidity index (CCI) and Acute Physiology Score (APS) III were utilized to calculate disease severity scores. This was done by assessing comorbidity, laboratory parameters, vital signs, and treatment measures.

### Outcomes

The main result was the mortality rate during hospitalization. The mortality rate in the hospital was determined by the flag indicating patient expiration in MIMIC-IV. The secondary outcomes were the length of stay in the hospital or ICU and the incidence of AKI. AKI may be diagnosed if any of the following criteria are met: (1) a surge in serum creatinine of more than 26.5 μmol/L (0.3 mg/dL) within 48 h; (2) a rise in serum creatinine exceeding 1.5 times the baseline level; (3) a sustained urine output of less than 0.5 ml/(kg h) for more than 6 h^21^.

### Statistical analysis

The sample size for this study was determined using Power Analysis and Sample Size Software (PASS 2021). Previous literature indicated that the ICU mortality rate was estimated to be 10% in patients with AHF^22^, and we anticipated a 20% difference between the two groups. With 90% power to detect a difference in in-hospital mortality between the two groups using a one-sided Z-test with pooled variance, a total sample size of 1168 was deemed necessary to evaluate the study’s primary endpoint^23^.

The mean and standard deviation were used to represent continuous variables that followed a normal distribution, whereas the median with interquartile range was used for variables with a nonnormal distribution. The Kruskal–Wallis test and ANOVA test wereused to conduct group comparisons. Counts and percentages were used to present categorical variables and compared using the Pearson chi-square test. Cox proportional hazards regression analysis and linear regression analysis were utilized to evaluate the association between the Na/Cl ratio and in-hospital mortality, AKI, and length of stay in hospital or ICU. Significant variables associated with endpoints were identified using univariate analysis (*P* < 0.05). Following that, variables with statistical significance were incorporated as covariates in the multivariate analysis. In accordance with the STROBE statement, initial analyses were conducted without any adjustments. Additional adjustments were made for various factors, including age, sex (in a minimally adjusted model), history of myocardial infarction, COPD, diabetes, chronic kidney disease, liver disease, CCI, APSIII, heart rate, mean blood pressure, glucose, serum calcium, serum potassium, blood urea nitrogen, eGFR, WBC, hemoglobin, platelets, and bicarbonate (in a fully adjusted model). Kaplan–Meier curves were used to illustrate the cumulative risk of mortality and AKI across the three tertiles of the NA/Cl ratio. To further test the predictive value of Na/Cl ratio, we performed receiver operator characteristic (ROC) curve analysis for in-hospital mortality based on serum sodium levels, serum chloride levels, Na/Cl ratio. The GWTG-HF score is a scoring system developed by the American Heart Association to assess in-hospital mortality rates in patients with AHF, with serum sodium levels being a key component of the score^24^. We generated ROC curves for the GWTG-HF score and for the ROC curve using the Na/Cl ratio as a substitute for serum sodium. To evaluate the relationship between mortality rates and the NA/Cl ratio, subgroup analysis was performed on the subgroups of hyponatremia and hypochloremia. The statistical analysis was conducted with the utilization of R software (R Foundation, Vienna, Austria) and Free Statistics software (version 1.8). Statistical significance was attributed to a significance level below *P* < 0.05.

### Sensitivity analysis

To conduct a sensitivity analysis, the robustness of the multiple regression findings through the utilization of tertiles for the Na/Cl ratio were additionally examined. A median was assigned as a continuous variable to each tertiles of the Na/Cl ratio, followed by conducting linear tests^25^. To assess the possibility of unmeasured confounding factors between the Na/Cl ratio and in-hospital mortality, the E-value was calculated. In our studies, the E-value serves as a statistical metric to assess the extent of unmeasured confounding variables that could potentially invalidate the observed correlations between the Na/Cl ratio and in-hospital mortality^26^. The researchers conducted subgroup analyses and examined interactions between subgroups using the likelihood ratio test.

### Ethics approval and consent to participate

MIMIC IV is a publicly, available ICU database with anonymous patient information, approved ethical review and data sharing. Written informed consent for participation was not required for this study in accordance with the national legislation and the institutional requirements.

## Results

### Participant selection

This study included a total of 7844 patients with AHF after they were screened based on the specified criteria. The flowchart of the case selection is depicted in Fig. 1.

### Baseline characteristics of participants

Table 1 presents the baseline characteristics of the participants categorized into three groups (Q1, Q2, and Q3) based on the admission Na/Cl ratio tertiles. Group Q1 included 2607 cases with an Na/Cl ratio range of 0.95–1.33, group Q2 included 2606 cases with an Na/Cl ratio range of 1.33–1.38, and group Q3 included 2631 cases with an Na/Cl ratio range of 1.38–1.75. The characteristics of the individuals who were enrolled are presented in Table 1. The average age of the patients included was 73.0, with a deviation of 13.7 from the mean. No statistically significant distinctions were noted among the three groups regarding the combined occurrence of systolic HF and diastolic HF, body temperature, WBC, NT-proBNP, presence of liver disease, or ICU length of stay (*P* > 0.05). Nevertheless, the prevalence of accompanying conditions, such as COPD, chronic kidney disease, diabetes, specific types of AHF, such as diastolic HF, and disruption in sodium or chloride levels, such as hyponatremia and hypochlorhydria, increased with increasing Na/Cl ratios (all *P* < 0.001). As the Na/Cl ratios increased, there was a decrease in the occurrence of systolic HF and hyperchloremia. Additionally, the utilization of MV, PCI, and diuretics showed a significant increase (*P* < 0.001). APS III scores, commonly used tools for evaluating the seriousness of critically ill individuals, including those with AHF, showed a significant correlation with elevated Na/Cl ratios (*P* < 0.001). With an increase in the Na/Cl ratio, patients exhibited heightened volatility in vital signs, an increased occurrence of kidney dysfunction, decreased SaO_2_ levels, and increased blood glucose levels (all *P* < 0.001).

**Table 1:** Baseline characteristics of the study participants.

Quartile of Na/Cl ratio	Total	Q1	Q2	Q3	*P*
	0.95–1.75	0.95–1.33	1.33–1.38	1.38–1.75	
N	n = 7844	n = 2607	n = 2606	n = 2631	
Demographics					
Sex, n (%)					0.004
Male	4205 (53.6)	1404 (53.9)	1454 (55.8)	1347 (51.2)	
Female	3639 (46.4)	1203 (46.1)	1152 (44.2)	1284 (48.8)	
Age, n (%)	73.0 ± 13.7	72.8 ± 13.5	73.7 ± 13.6	72.4 ± 13.8	0.002
Classification of AHF, n (%)					
Systolic heart failure	3072 (39.2)	1082 (41.5)	1061 (40.7)	929 (35.3)	< 0.001
Diastolic heart failure	3750 (47.8)	1210 (46.4)	1187 (45.5)	1353 (51.4)	< 0.001
Combined both	1006 (12.8)	308 (11.8)	355 (13.6)	343 (13)	0.137
History of disease, n (%)					
Myocardial infarction	2644 (33.7)	843 (32.3)	977 (37.5)	824 (31.3)	< 0.001
Cerebrovascular disease	993 (12.7)	320 (12.3)	379 (14.5)	294 (11.2)	< 0.001
COPD	2934 (37.4)	834 (32)	936 (35.9)	1164 (44.2)	< 0.001
Liver disease	705 (9.0)	261 (10)	218 (8.4)	226 (8.6)	0.079
Chronic kidney disease	3085 (39.3)	931 (35.7)	944 (36.2)	1210 (46)	< 0.001
Diabetes	2351 (30.0)	717 (27.5)	785 (30.1)	849 (32.3)	< 0.001
Hypertension	7437 (94.8)	2475 (94.9)	2469 (94.7)	2493 (94.8)	0.939
Scoring system					
CCI	7.3 ± 2.5	7.0 ± 2.5	7.3 ± 2.5	7.6 ± 2.5	< 0.001
APSIII	52.0 ± 21.7	51.8 ± 22.7	50.2 ± 21.6	53.8 ± 20.6	< 0.001
Vital signs at presentation					
Heart rate (beats/minute)	84.8 ± 16.4	84.1 ± 15.6	84.4 ± 16.7	85.7 ± 16.9	0.001
MBP (mmHg)	76.3 ± 10.6	75.0 ± 9.8	76.9 ± 10.7	76.9 ± 11.0	< 0.001
RR (times/minute)	20.0 ± 3.8	19.5 ± 3.7	20.1 ± 3.8	20.4 ± 3.8	< 0.001
Temperature (℃)	36.8 ± 0.5	36.8 ± 0.5	36.8 ± 0.5	36.8 ± 0.5	0.517
SaO_2_ (%)	96.6 ± 2.1	97.1 ± 2.0	96.6 ± 2.1	96.1 ± 2.2	< 0.001
Laboratory findings					
White cell count (10^9^/L)	9.3 (6.9, 12.6)	9.4 (6.9, 12.9)	9.2 (6.8, 12.4)	9.2 (6.9, 12.4)	0.22
Hemoglobin (g/dL)	11.2 ± 2.1	11.0 ± 1.9	11.4 ± 2.2	11.3 ± 2.3	< 0.001
Platelets (K/μL)	212 (160, 278)	197 (148, 258)	216 (165, 278)	221 (169, 294)	< 0.001
Glucose (mmol/L)	8.4 (6.7, 11.3)	7.7 (6.4, 10.2)	8.5 (6.8, 11.3)	9.0 (7.1, 12.4)	< 0.001
Serum creatinine (mg/dl)	1.6 ± 1.5	1.4 ± 1.1	1.5 ± 1.2	2.0 ± 2.0	< 0.001
Calcium (mmol/L)	8.7 ± 1.1	8.5 ± 1.7	8.7 ± 0.7	8.9 ± 0.8	< 0.001
Potassium (mmol/L)	4.0 ± 0.6	4.1 ± 0.6	4.0 ± 0.6	3.9 ± 0.6	< 0.001
Sodium (mmol/L)	136.3 ± 5.3	137.0 ± 4.8	136.5 ± 4.6	135.4 ± 6.2	< 0.001
Chloride (mmol/L)	100.4 ± 6.5	105.9 ± 4.4	100.8 ± 3.6	94.5 ± 5.6	< 0.001
Urinary sodium (mEq/L) (n = 3604)	39.0 (23.0, 66.2)	41.0 (24.0, 70.0)	40.0 (22.8, 66.0)	37.0 (22.0, 4.0)	0.04
Urinary chloride (mEq/L) (n = 2600)	37.0 (20.0, 73.0)	37.0 (20.0, 75.0)	35.0 (18.5, 4.5)	37.0 (20.0, 9.0)	0.305
Blood urea nitrogen (mmol/L)	5.0 (17.0, 40.0)	2.0 (15.0, 35.0)	4.0 (16.0, 37.0)	9.0 (19.0, 48.0)	0.001
Albumin (g/dl) (n = 3187)	3.3 ± 0.6	3.1 ± 0.6	3.3 ± 0.6	3.4 ± 0.6	0.001
Bicarbonate (mmol/L)	22.3 ± 5.2	20.1 ± 3.8	22.0 ± 4.1	24.9 ± 6.0	< 0.001
NT-proBNP (pg/mL)	3808.0 (1389.2, 10,101.8)	3817.0 (1399.0, 9978.0)	3654.5 (1370.5, 9428.0)	3962.0 (1413.0, 11,022.0)	0.177
eGFR	67.0 (38.9, 91.7)	73.1 (44.4, 94.6)	69.8 (43.0, 92.2)	56.8 (29.7, 86.8)	< 0.001
NACL ratio	1.4 ± 0.1	1.3 ± 0.0	1.4 ± 0.0	1.4 ± 0.0	< 0.001
Type of imbalance of sodium or chloride, n (%)					
Hyponatremia	1913 (24.4)	516 (19.8)	580 (22.3)	817 (31.1)	< 0.001
Hypochlorhydria	1525 (19.4)	32 (1.2)	202 (7.8)	1291 (49.1)	< 0.001
Hypernatremia	133 (1.7)	63 (2.4)	31 (1.2)	39 (1.5)	< 0.001
Hyperchloremia	1220 (15.6)	1133 (43.5)	84 (3.2)	3 (0.1)	0.002
Treatment and medications, n (%)					
PCI	700 (8.9)	242 (9.3)	257 (9.9)	201 (7.6)	0.014
MV	1658 (21.1)	669 (25.7)	503 (19.3)	486 (18.5)	< 0.001
Use of diuretic	4515 (57.6)	1375 (52.7)	1516 (58.2)	1624 (61.7)	< 0.001
Outcomes					
In-hospital mortality, n (%)	776 (9.9)	209 (8)	243 (9.3)	324 (12.3)	< 0.001
Los hospital (days)	11.8 ± 10.2	11.9 ± 10.0	11.3 ± 9.2	12.4 ± 11.3	< 0.001
Los ICU (days)	4.5 ± 5.5	4.6 ± 5.4	4.4 ± 5.4	4.5 ± 5.8	0.475
AKI in 2 days	5622 (71.7)	1943 (74.5)	1786 (68.5)	1893 (71.9)	< 0.001
AKI in 7 days	6030 (76.9)	2056 (78.9)	1926 (73.9)	2048 (77.8)	< 0.001

*COPD* Chronic obstructive pulmonary disease, *CCI* Charlson comorbidity index, *APSIII* Simplified acute physiology score III, *MBP* Mean blood pressure, *RR* Respiratory rate, *SaO*_*2*_ Oxygen saturation of blood, *NT-proBNP* N-terminal pro-brain natriuretic peptide, *eGFR* Estimated glomerular filtration rate, *PCI* Percutaneous coronary intervention, *MV* Mechanical ventilation, *Los hospital* Length of stay in hospital, *Los ICU* Length of stay in ICU, *AKI* Acut kidney injury.

### Outcomes

During the initial hospital stay, a total of 776 individuals (9.9%) passed away. It was observed that patients who experienced an elevation in their Na/Cl ratio also had a proportional rise in the mortality rate (8% vs. 9.3% vs. 12.3%; *P* < 0.001), length of the time spent in the hospital, and incidence of AKI.

### Na/Cl ratio and outcomes

**Figure 2:**
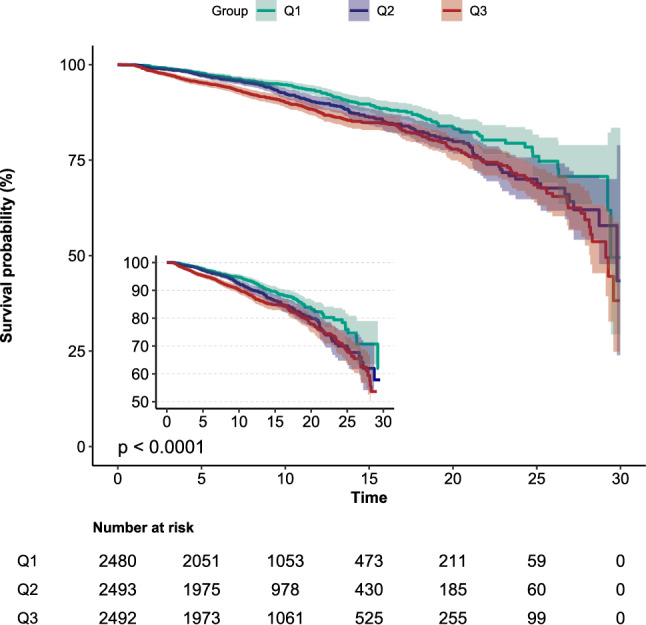
Kaplan–Meier survival curves for in-hospital mortality of patients with AHF depending on the Na/Cl ratio.

**Figure 3:**
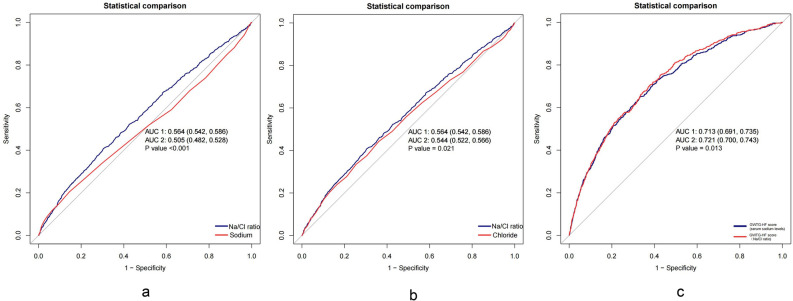
(**a**) Comparison of ROC curve for in-hospital mortality of AHF patients between Na/Cl ratio and serum sodium level. (**b**) Comparison of ROC curve for in-hospital mortality of AHF patients between Na/Cl ratio and serum chloride level. (**c**) Comparison of ROC curve for in-hospital mortality of AHF patients between primary GWTG-HF score and adjusted GWTG-HF score (replace serum sodium levels with Na/Cl ratio).

During hospitalization, the high Na/Cl ratio group demonstrated a lower survival rate than the low Na/Cl ratio group, as indicated by Kaplan–Meier analysis (*P* < 0.001) (Fig. 2). However, there was no difference in the incidence of AKI among the three groups (*P* = 0.76) (Supplementary Fig. 1). The mortality rate was greater in the hypochloremia group than in the non-hypochloremia group (*P* = 0.011) (Supplementary Fig. 2a), but there was no statistically significant difference in mortality rate between the hyponatremia group and the non-hyponatremia group (*P* = 0.53) (Supplementary Fig. 2b). The likelihood of developing AKI was higher in the hypochloremia group and the hyponatremia group compared to the non-hyponatremia group or non-hypochloremia group (*P* < 0.001 for all) (Supplementary Fig. 2c, 2d). The dependent variable chosen was the indicator of in-hospital mortality, and a univariate analysis was conducted to investigate the correlation between this variable and the independent variables. The findings showed that various factors, including age, myocardial infarction, cerebrovascular disease, chronic kidney disease, liver disease, CCI, APSIII, mean blood pressure, heart rate, body temperature, SaO2, WBC, serum creatinine, BUN, sodium, potassium, calcium, chloride, eGFR, NT-proBNP, bicarbonate, hypochlorhydria, hypernatremia, use of diuretics and MV, were all linked to mortality during hospitalization (Table 2). In the unadjusted Cox hazard regression model, the Na/Cl ratio demonstrated a significant association with an increased risk of in-hospital mortality as a continuous variable (HR = 1.30, 95% CI 1.18–1.43). After additional modification for all possible confounding factors, the associations were slightly strengthened but still significant, showing a hazard ratio of 1.34 (95% CI 1.2–1.5) (Table 3). In the fully adjusted model, participants in the high tertile groups of the Na/Cl ratio had a 54% higher chance of in-hospital mortality than those in the low tertile groups. This increase in risk was statistically significant, with a *P* value for the trend of less than 0.001 (Table 3). The relationships between Na/Cl ratio and secondary outcomes indicate that as the Na/Cl ratio increases, the length of hospital stay increases, whereas the ICU stay decreases. There was no significant correlation between AKI occurrence and Na/Cl ratio (Supplementary Table 2). ROC curve analysis was conducted to evaluate the potential prognostic significance of the Na/Cl ratio in patients with AHF. Compare with the individual serum sodium or serum chloride levels, the Na/Cl ratio exhibited greater sensitivity in predicting in-hospital mortality in AHF patients (0.564 vs. 0.544 and 0.505, respectively) (Fig. 3a, b). The C statistic for the GWTG-HF score (utilizing Na/Cl ratio instead of serum sodium levels) was superior to that of the original GWTG-HF score (0.721 vs. 0.713) (Fig. 3c).

**Table 2:** Univariate analysis for in-hospital mortality.

Covariate	*HR* (95% CI)	*P*
Demographics		
Gender		
Male	1.0	
Female	0.98 (0.85, 1.13)	0.807
Age	1.04 (1.04, 1.05)	< 0.001
Classification of AHF		
Systolic heart failure	1.37 (1.19, 1.57)	< 0.001
Diastolic heart failure	0.73 (0.64, 0.85)	< 0.001
Combined both	0.9914 (0.8012, 1.2267)	0.937
History of disease		
Myocardial infarction	1.51 (1.31, 1.74)	< 0.001
Cerebrovascular disease	1.29 (1.07, 1.54)	0.006
Chronic pulmonary disease	0.99 (0.85, 1.14)	0.875
Liver disease	1.29 (1.06, 1.58)	0.012
Diabetes	0.88 (0.75, 1.03)	0.112
Hypertension	0.96 (0.7, 1.32)	0.815
Chronic kidney disease	1.42 (1.23, 1.64)	< 0.001
Scoring system		
Charlson comorbidity index	1.15 (1.12, 1.18)	< 0.001
APSIII	1.02 (1.02, 1.03)	< 0.001
Vital signs at presentation		
Heart rate	1.0033 (0.9991, 1.0075)	0.119
MBP	0.97 (0.97, 0.98)	< 0.001
Respiration rate	1.06 (1.04, 1.08)	< 0.001
Temperature	0.65 (0.58, 0.74)	< 0.001
SaO_2_	0.91 (0.89, 0.94)	< 0.001
Laboratory findings		
White blood cell	1.02 (1.01, 1.02)	< 0.001
Hemoglobin	0.98 (0.95, 1.02)	0.284
Platelet	1.0003 (0.9998, 1.0009)	0.264
Calcium	1.04 (1, 1.07)	0.034
Potassium	1.21 (1.13, 1.3)	< 0.001
Chloride	0.99 (0.98, 1)	0.007
Sodium	1.02 (1, 1.03)	0.028
Urinary sodium	0.9927 (0.9891, 0.9963)	0.012
Urinary chloride	0.9954 (0.9916, 0.9991)	< 0.001
Albumin	0.79 (0.68, 0.91)	0.002
Glucose	1.0012 (1.0007, 1.0017)	< 0.001
NA/CL ratio (Per 0.1)	1.3 (1.18 ~ 1.43)	< 0.001
Serum creatinine	1.09 (1.05, 1.13)	< 0.001
Blood urea nitrogen	1.01 (1.01, 1.02)	< 0.001
eGFR	0.99 (0.99, 0.99)	< 0.001
NT-proBNP	1 (1, 1)	< 0.001
Type of imbalance of sodium or chloride		
Hyponatremia	1.05 (0.9, 1.23)	0.535
Hypochlorhydria	1.23 (1.05, 1.45)	0.011
Hyperchloremia	0.83 (0.68, 1.03)	0.086
Hypernatremia	2.13 (1.46, 3.11)	< 0.001
Treatment and medications		
PCI	0.99 (0.74, 1.31)	0.931
MV	1.38 (1.19, 1.61)	< 0.001
Use of diuretic	0.56 (0.49, 0.65)	< 0.001

*HR* Hazard ratio; *CI* Confidence interval.

**Table 3:** Association between Na/Cl ratio and in-hospital mortality in multiple regression model.

		Model I		Model II		Model III		Model IV	
		HR (95% CI)	*P* value	HR (95% CI)	*P* value	HR (95% CI)	*P* value	HR (95% CI)	*P* value
Na/Cl ratio^a^									
In-hospital mortality	1.30 (1.18–1.43)	< 0.001	1.35 (1.22–1.49)	< 0.001	1.44 (1.31–1.59)	< 0.001	1.34 (1.2–1.5)	< 0.001	
Na/Cl ratio tertiles									
In-hospital mortality	Q1	1 (Ref)		1 (Ref)		1 (Ref)		1 (Ref)	
	Q2	1.25 (1.04–1.5)	0.019	1.22 (1.02–1.47)	0.033	1.36 (1.13–1.64)	0.001	1.3 (1.07–1.57)	0.008
	Q3	1.46 (1.23–1.74)	< 0.001	1.51 (1.27–1.8)	< 0.001	1.72 (1.44–2.06)	< 0.001	1.54 (1.26–1.88)	< 0.001
	*P* fortrend		< 0.001		< 0.001		< 0.001		< 0.001

^a^NA/CL ratio was entered as continuous variable per 0.1 increase.

Model I: didn’t adjusted for confounders.

Model II: adjusted for age, sex.

Model III: Model II + myocardial infarction, COPD, diabetes, chronic kidney disease, liver disease, CCI, APSIII.

Model IV: Model III + heart rate, mean blood pressure, glucose, blood urea nitrogen, serum calcium, serum potassium, eGFR, white cell count, hemoglobin, platelets, bicarbonate.

### Sensitivity analysis and subgroup analysis

**Figure 4:**
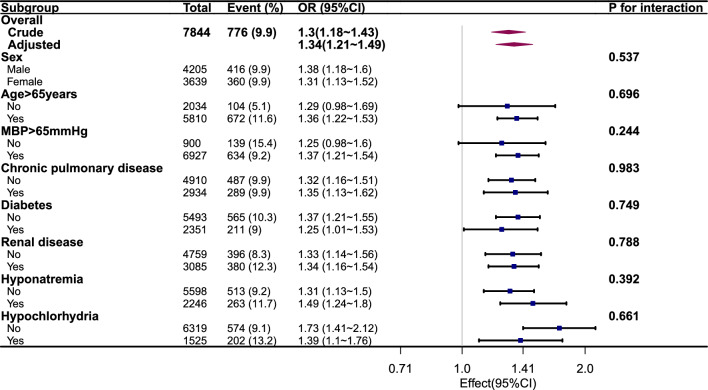
Risk of primary outcome for Na/Cl ratio in different subgroups of patients. Hazard ratio (HR) was adjusted for age, sex, myocardial infarction, COPD, diabetes, renal disease, liver disease, CCI, APSIII, heart rate, mean blood pressure, glucose, blood urea nitrogen, serum calcium, serum potassium, eGFR, white cell count, hemoglobin, platelets, bicarbonate.

The linear trend tests of the four models indicated that there was a significant association between higher tertile groups of the Na/Cl ratio and an elevated risk of in-hospital mortality. In Table 3, the *P* values for the trend test were less than 0.001 for all Model I to Model IV. To assess the unmeasured confounders, the E-value was computed as a component of the sensitivity analysis. The findings indicated that the primary results remained consistent unless there was a strong positive correlation between unmeasured confounders and the Na/Cl ratio and in-hospital mortality (OR ≥ 2.015). To evaluate the effect of the ratio of Na/Cl on in-hospital death in different subgroups, subgroup analyses were conducted. The findings indicated a positive correlation in specific subcategories, without any significant interactions among the groups (interaction *P* > 0.05) (Fig. 4).

## Discussion

This study retrospectively examined a group of critically ill patients with AHF in the ICU to explore the correlation between the ratio of Na/Cl and the mortality rate during hospitalization. The study’s results showed a notable and favorable connection between the Na/Cl ratio and in-hospital death among ICU patients with AHF, even after thoroughly accounting for potential confounding variables in different models. To confirm our findings, sensitivity analyses by dividing the Na/Cl ratio into three equal groups were performed, and the observed correlation remained strong and statistically significant (with the initial group used as the baseline, the hazard ratio steadily increased across the groups, resulting in a trend *P* value less than 0.001 in the fully adjusted model). The results underscore the importance of taking into account the ratio of sodium to chloride as a predictive indicator in critically ill individuals with AHF, offering valuable perspectives for categorizing risk and tailoring treatment approaches.

Our study sought to assess the frequency of electrolyte imbalances in critically ill patients with AHF in the ICU. These imbalances, commonly seen in patients with HF, may be attributed to the use of diuretics causing electrolyte depletion or fluid therapy resulting in dilution of electrolytes in the blood. According to our research, over 50% of the patients displayed irregularities in the levels of sodium or chloride in their blood. In particular, 28.6% of the patients exhibited hyponatremia, whereas hypochloremia was found in 19.4% of instances. Hypernatremia and hyperchloremia occurred in 1.7% and 15.6% of patients, respectively. Notably, among the electrolyte imbalances observed in patients with AHF, hyponatremia and hypochloremia were the most common. This can be attributed to the maladaptive response of the activated neurohormonal system during the progression of HF, leading to increased reabsorption of free water and subsequent electrolyte dilution, including sodium and chloride^27^. AHF is typically associated with elevated levels of BNP. BNP is released in response to increased cardiac wall tension and volume overload. These peptides stimulate natriuresis, promoting the excretion of sodium in the urine by acting on the kidneys to enhance sodium excretion^28^. Increased sodium excretion results in hyponatremia in the bloodstream. In reaction to elevated sodium excretion, there is compensatory reabsorption of chloride ions in the renal tubules, leading to hyperchloremia in the blood^29^. As a result, the release of natriuretic peptides in patients with HF contributes to the development of hyponatremia and hyperchloremia through their influence on sodium and chloride regulation in the kidneys^30^. Furthermore, the intensive and prolonged use of diuretics in these patients, aimed at alleviating congestion, may contribute to electrolyte disturbances by limiting electrolyte reabsorption^31,32^. Ultimately, these factors contribute to the reduction in serum sodium and chloride concentrations.

The presence of hyponatremia in individuals with AHF is seen as an indication of the severity of the disease and is a sign of higher rates of occurrence and death. Extensive research has shown that even mild hyponatremia is a strong predictor of adverse events, including worsened survival, increased readmission rates, prolonged hospital stays, increased utilization of hospital resources, and higher costs^33,34^. Nevertheless, the significance of serum chloride, a prevalent primary anion in the basic metabolic panel, was frequently disregarded until a 2007 study linked decreased serum chloride levels to a heightened mortality risk in HF patients^35^. Since then, multiple studies have consistently demonstrated a strong independent relationship between hypochloremia and increased mortality risk in both acute and chronic HF, regardless of serum sodium levels^36–39^. In our investigation, it was found that hypochloremia was positively correlated with in-hospital mortality among patients, while hyponatremia did not show a similar association. A recent study incorporating serum chloride into the existing HF prognostic model significantly increased the area under the receiver operating characteristic curve^40^. Another study demonstrated that changes in serum chloride levels can help identify patients with poor prognoses and determine appropriate treatment strategies^41^. These findings are consistent with our research results. These results indicate that chloride ions might have a more significant influence on the prognosis of individuals with HF than sodium ions.

It is important to note that decreases in serum sodium concentration do not occur in isolation; to maintain electroneutrality, anions such as chloride ions or bicarbonate must change in parallel with sodium. Consequently, hyponatremia and hypochloremia often coexist. Traditionally, serum chloride has been considered a passive change in response to alterations in serum sodium concentration. However, emerging evidence suggests that the opposite may be true. Studies suggest that alterations in plasma volume, vasopressin release, and the renin–angiotensin–aldosterone system (RAAS) during exacerbation of HF are predominantly influenced by chloride ions in the bloodstream, as opposed to levels of sodium in the bloodstream^42^.

Although natriuretic hormone antagonists have been shown to significantly improve serum sodium levels, recent trials have yielded disappointing short-term and long-term effects^43,44^. This suggests that the impact of serum chloride may not have been adequately considered. While serum sodium and chloride generally change in tandem, lower serum chloride levels often occur concurrently with lower serum sodium levels, indicating more severe internal balance abnormalities^45^. However, in patients with HF, the changes in sodium and chloride may be imbalanced. During our investigation, a greater occurrence of low sodium levels in comparison to low chloride levels was noticed, and the effects of these two conditions on the outlook of patients with HF were found not to occur simultaneously. Hence, depending exclusively on serum levels of sodium or chloride for assessing the prognosis of individuals with HF might not offer a thorough evaluation. It is crucial to consider both factors in combination. Our study also demonstrated that in the GWTG-HF score, by fully considering the interaction between serum sodium and serum chloride, replacing serum sodium with NA/CL ratio resulted in greater predictive power.

To evaluate the outcome of patients with AHF, the Na/Cl ratio was employed in our study. The results indicated a direct relationship between the mortality rates of patients with AHF during their hospital stay and the Na/Cl ratio. The Q1 group, which had low levels of Na/Cl, showed the highest occurrence of low sodium (23.3%) and high chloride (43.5%). The Q3 group, which had high Na/Cl levels, showed the highest occurrence of low sodium (36.1%) and low chloride (49.1%) combination. The frequency and intensity of low chloride were greater than those of low sodium, suggesting that serum chloride might have a more significant influence on the prognosis of individuals with HF than serum sodium.

Evaluating the outlook of individuals diagnosed with AHF by utilizing the Na/Cl ratio enables a thorough assessment of the collective impact of sodium and chloride levels in the bloodstream. One study investigated the impact of serum sodium and serum chloride on the prognosis of patients with acute decompensated heart failure (ADHF). The results showed a linear relationship between serum sodium levels and in-hospital mortality rates, with in-hospital mortality rates increasing as serum sodium levels gradually decreased. In contrast, serum chloride exhibited a U-shaped relationship, with the lowest in-hospital mortality rate observed at serum chloride levels of 101–105 mmol/L^46^. Unfortunately, the study did not explore the combined effects of serum sodium and serum chloride. In the Chinese population, a study discovered a U-shaped correlation between the ratio of Na/Cl and rates of readmission for HF, which shows slight variation compared to our study^47^. This disparity may be attributed to differences in the racial and demographic characteristics of the study populations. They examined a cohort of Chinese patients, whereas our study focused on a population of critically ill patients with AHF from Western countries. These patients had more severe conditions and a higher prevalence of comorbidities, resulting in a higher incidence and severity of low sodium and chloride. Low levels of sodium and chloride serve as markers of disease severity.

The survival rate of patients with chronic HF is significantly and independently linked to the levels of chloride in the serum. Hypochloremia contributes significantly to the risks associated with hyponatremia. This implies that the negative outlook in individuals with HF caused by hyponatremia is partly affected by hypochloremia^11,14^. The significance of chloride in maintaining fluid balance, activating neurohormones, and countering the effects of diuretics has been highlighted in recent studies, all of which are considered crucial elements in the development and advancement of HF^45,47^. Hence, the level of chloride in the blood can be a crucial determinant and shed light on numerous hazards linked to sodium levels in the blood.

In a large sample population, the influence of the ratio of sodium to chloride on the prognosis of patients with AHF was examined, considering the interplay between these two elements. The results offer valuable perspectives on the predictive consequences of the interaction between sodium and chloride, enhancing comprehension of their reciprocal impacts. Furthermore, the assessment of the Na/Cl ratio continues to be significant for medical professionals in enhancing patient results and implementing better and more focused treatment strategies, such as electrolyte control. This fresh viewpoint presents valuable observations for clinical approaches to AHF while also supplying crucial data for additional investigation into the fundamental mechanisms of sodium and chloride interactions. When evaluating the prognosis of patients with AHF, it is important to take into account both factors.

Nevertheless, it is crucial to recognize the limitations of our research. First, it was a retrospective study, and although numerous variables were included, there may still be potential confounding factors that were not accounted for. As a cohort study, it cannot establish a causal relationship. Additionally, we failed to gather information regarding the fluctuation of the ratio between sodium and chloride, which can be affected by the usage of diuretics and potentially affect the mortality rates of patients. Furthermore, although our study had a substantial sample size, the findings are only applicable to a specific group of severely ill individuals with AHF in the United States. To establish its applicability to other populations, additional research is needed. Further investigation is needed to explore the mechanisms that contribute to the effect of the Na/Cl ratio on mortality rates during hospitalization in critically ill patients with AHF.

## Conclusion

To summarize, our research shows a direct link between the ratio of sodium to chloride and the rates of mortality during hospitalization in patients with AHF who are critically ill. This emphasizes the significance of taking into account the interplay between sodium and chloride levels in the bloodstream. Both factors should be carefully considered in the management of AHF.

### Supplementary Information

Supplementary Figure 1.

Supplementary Figure 2.

Supplementary Table 1.

Supplementary Table 2.

## Data Availability

The clinical data used to support the findings of this study were supplied by Monitoring in Intensive Care Database IV version 2.0 (MIMIC-IV v.2.0) (https://physionet.org/content/mimic-iv-demo/2.2/). Although the database is publicly and freely available, researchers must complete the National Institutes of Health’s web-based course known as Protecting Human Research Participants to apply for permission to access the database. Data are available to researchers on request for purposes of reproducing the results or replicating the procedure by directly contacting the corresponding author.

## Abbreviations

AHF: Acute heart failure
HF: Heart failure
COPD: Chronic obstructive pulmonary disease
CCI: Charlson comorbidity index
APSIII: Simplified acute physiology score III
MBP: Mean blood pressure
RR: Respiratory rate
SaO_2_: Oxygen saturation of blood
NT-proBNP: N-terminal pro-brain natriuretic peptide
eGFR: Estimated glomerular filtration rate
PCI: Percutaneous coronary intervention
MV: Mechanical ventilation
Los hospital: Length of stay in hospital
Los ICU: Length of stay in ICU
AKI: Acute kidney injury
HR: Hazard ratio
CI: Confidence interval
