# Supplementary material for: Positive association between sodium-to-chloride ratio and in-hospital mortality of acute heart failure
Source: Sci Rep. 2024 Apr 3;14:7846. doi: 10.1038/s41598-024-58632-4 (PMC10991295; doi:10.1038/s41598-024-58632-4)
Supplement: Supplementary file 1 — Supplementary Figure 1. [file 41598_2024_58632_MOESM1_ESM.pdf]

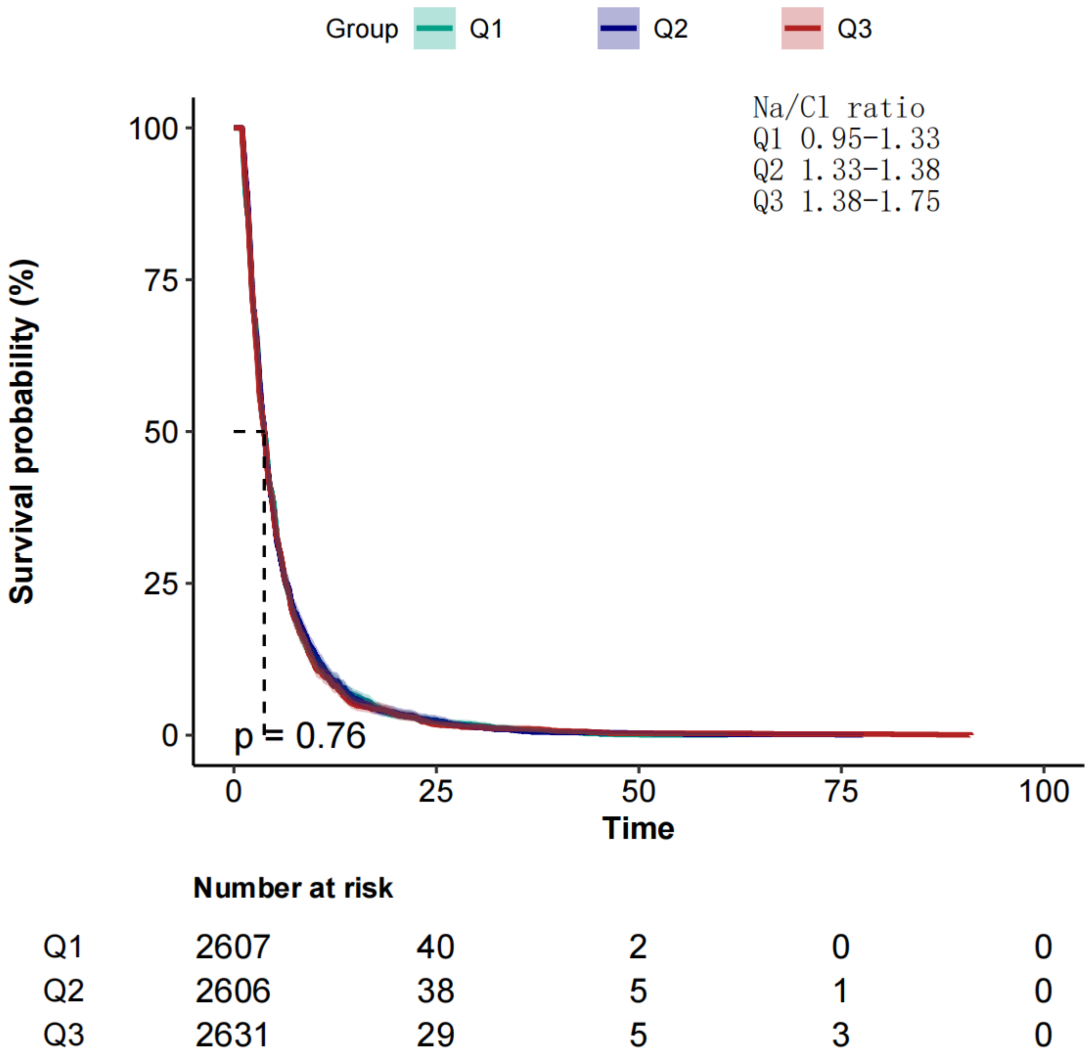

Supplementary Figure 1 Kaplan–Meier survival curves for likelihood of developing AKI in patients with AHF according to the Na/Cl ratio
