# Supplementary material for: Positive association between sodium-to-chloride ratio and in-hospital mortality of acute heart failure
Source: Sci Rep. 2024 Apr 3;14:7846. doi: 10.1038/s41598-024-58632-4 (PMC10991295; doi:10.1038/s41598-024-58632-4)
Supplement: Supplementary file 2 — Supplementary Figure 2. [file 41598_2024_58632_MOESM2_ESM.pdf]

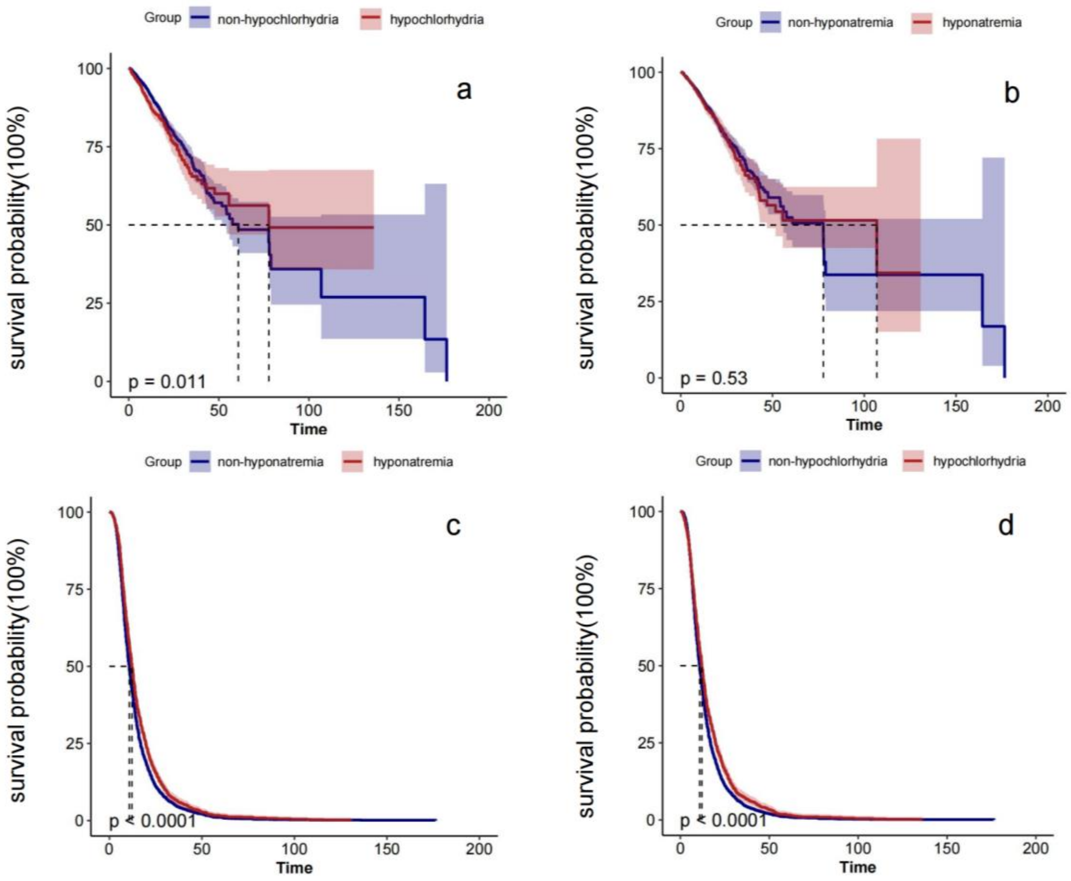

Supplementary Figure 2a Kaplan–Meier survival curves for in-hospital mortality of patients with AHF depending on the serum chloride level **2b** Kaplan–Meier survival curves for in-hospital mortality of patients with AHF depending on the serum sodium level **2c** Kaplan–Meier survival curves for likelihood of developing AKI of patients with AHF depending on the serum sodium level **2d** Kaplan–Meier survival curves for likelihood of developing AKI of patients with AHF depending on the serum chloride level.
