# Supplementary material for: Positive association between sodium-to-chloride ratio and in-hospital mortality of acute heart failure
Source: Sci Rep. 2024 Apr 3;14:7846. doi: 10.1038/s41598-024-58632-4 (PMC10991295; doi:10.1038/s41598-024-58632-4)
Supplement: Supplementary file 3 — Supplementary Table 1. [file 41598_2024_58632_MOESM3_ESM.docx]

**Supplementary Table 1** ICD codes and their corresponding diagnostic translations included in the study.

| ICD code | ICD version | Diagnose |
| --- | --- | --- |
| 42821 | 9 | Acute systolic heart failure |
| 42823 | 9 | Acute on chronic systolic heart failure |
| 42831 | 9 | Acute diastolic heart failure |
| 42833 | 9 | Acute on chronic diastolic heart failure |
| 42841 | 9 | Acute combined systolic and diastolic heart failure |
| 42843 | 9 | Acute on chronic combined systolic and diastolic heart failure |
| I5021 | 10 | Acute systolic (congestive) heart failure |
| I5023 | 10 | Acute on chronic systolic (congestive) heart failure |
| I5031 | 10 | Acute diastolic (congestive) heart failure |
| I5033 | 10 | Acute on chronic diastolic (congestive) heart failure |
| I5041 | 10 | Acute combined systolic (congestive) and diastolic (congestive) heart failure |
| I5043 | 10 | Acute on chronic combined systolic (congestive) and diastolic (congestive) heart failure |
| I50811 | 10 | Acute right heart failure |
| I50813 | 10 | Acute on chronic right heart failure |
