# Supplementary material for: Positive association between sodium-to-chloride ratio and in-hospital mortality of acute heart failure
Source: Sci Rep. 2024 Apr 3;14:7846. doi: 10.1038/s41598-024-58632-4 (PMC10991295; doi:10.1038/s41598-024-58632-4)
Supplement: Supplementary file 4 — Supplementary Table 2. [file 41598_2024_58632_MOESM4_ESM.docx]

**Supplementary Table 2.** Relationship between Na/Cl ratio and secondary outcomes

| Outcomes | Non-adjusted | | Full-adjusted | |
| --- | --- | --- | --- | --- |
|  | Coefficient/HR  (95%CI) | *p* | Coefficient/HR  (95%CI) | *P* |
| Los hospital | 0.29* (-0.05~0.64) | 0.092 | 0.69* (0.27~1.1) | 0.001 |
| Los ICU | -0.07*(-0.26~0.11) | 0.429 | -0.23*(-0.44~-0.02) | 0.036 |
| AKI in 2 days | 0.92(0.85~0.99) | 0.022 | 0.97(0.87~1.07) | 0.542 |
| AKI in 7 days | 0.96(0.88~1.04) | 0.279 | 0.98(0.87~1.09) | 0.678 |

Note:Linear regression analysis and Cox regression analysis were used to analyze the outcomes with continuous and time-to-event data, respectively. '*' represents the coefficient value.Full-adjusted model was adjusted for age, sex, myocardial infarction, COPD, diabetes, renal disease,liver disease,CCI,APSIII, heart rate,mean blood pressure,glucose,serum calcium,serum potassium,eGFR,white cell count, hemoglobin, platelets, bicarbonate.
